# Effect of an Inflatable Colon on Colorectal Cancer Knowledge and Screening Intent Among Male Attendees at State Fairs in Two Midwestern States, 2023

**DOI:** 10.5888/pcd21.240020

**Published:** 2024-09-05

**Authors:** Ami E. Sedani, Kelly K. Rifelj, Malcolm S. Bevel, Cordero McCall, Mckenzi Rogalla, Lisa Laliberte, Kiara Ellis, Rebekah J. Pratt, Charles R. Rogers

**Affiliations:** 1 Institute for Health & Equity, Medical College of Wisconsin, Milwaukee; 2 Department of Medicine, Medical College of Georgia, Augusta; 3 Department of Orthopedic Surgery, Medical College of Wisconsin, Milwaukee; 4 MNGI Digestive Health, Minneapolis, Minnesota; 5 Masonic Cancer Center, Minneapolis, Minnesota; 6 Department of Family Medicine and Community Health, University of Minnesota, Minneapolis

## Abstract

**Introduction:**

Colorectal cancer (CRC) is the third most-diagnosed cancer among men and women in the US. This study aimed to evaluate the influence of an interactive inflatable colon exhibit on CRC knowledge and screening intent among men attending state fairs in 2 midwestern states.

**Methods:**

At the 2023 state fairs in 2 midwestern states, eligible participants (men aged 18–75 y who could speak and read English and resided in 1 of the 2 states) completed a presurvey, an unguided tour of the inflatable Super Colon, and a postsurvey. Primary outcomes were changes in knowledge (actual and perceived) and CRC screening intent from presurvey to postsurvey. We used χ^2^ tests to examine differences in survey results between the 2 sites and the association between demographic characteristics and behaviors (knowledge and intentions) before entering the Super Colon exhibit. We used the McNemar test to examine differences in presurvey to postsurvey distributions.

**Results:**

The study sample (N = 940) comprised 572 men at site A (60.8%) and 368 men at site B (39.2%). Except for 1 question, baseline CRC knowledge was relatively high. Greater perceived knowledge was inversely associated with greater actual knowledge. After touring the Super Colon, participants improved their actual knowledge of CRC prevention and self-perceived CRC knowledge. Most participants (95.4%) agreed that the Super Colon was effective for teaching people about CRC.

**Conclusion:**

These findings emphasize the role of community-based educational initiatives in encouraging CRC screening uptake and increasing research participation among men and affirm that the inflatable colon is as an effective educational tool for increasing CRC knowledge and encouraging early-detection screening behavior among men.

**Summary**

**What is already known on this topic?**

Knowledge and beliefs are factors that enable health behaviors such as participation in early-detection screening. Community education and outreach events are common approaches to fostering health-related knowledge and awareness.

**What is added by this report?**

Self-guided tours of an interactive, inflatable colon can be an effective and low-resource intervention to increase colorectal cancer knowledge and screening intent among men at state fairs.

**What are the implications for public health practice?**

State fairs and similar large recreational gatherings can reach populations who may not typically have easy access to or knowledge about cancer prevention and control services.

## Introduction

Colorectal cancer (CRC) is the third most-diagnosed cancer among men and women in the US and the second most common cause of cancer-related death in men and women combined (1), with both incidence and death rates higher among men (2). CRC rates among people younger than 50 years (ie, early-onset CRC) have increased by approximately 50% since the mid-1990s; thus, the US Preventive Services Task Force now recommends that average-risk adults start CRC screening at age 45 years (3,4). Moreover, the rate of early-onset CRC is 16% to 30% higher among men than women (5). Given the high incidence of and deaths from CRC among men, prioritizing CRC prevention efforts is a public health imperative.

The association of CRC knowledge and awareness with CRC screening uptake is well established (6–10). Community education and outreach events are common approaches to fostering health-related knowledge and awareness. Despite some data suggesting that special events — especially those that provide onsite screening services — may lead to increases in cancer screening, evidence to date is insufficient to demonstrate that such events are effective at boosting cancer screening (11).

An innovative resource, the inflatable colon — a super-sized model of the human colon through which visitors can walk–– is a tool for teaching about the digestive system and for engaging and educating people about CRC and other colon diseases. Multiple studies have demonstrated that the use of the inflatable colon can improve CRC-related knowledge among young adults, Hispanic people, African American men, and others (12–18). A giant inflatable colon was shown to offer (14) a promising community-level intervention focused on enhancing CRC screening and prevention through a novel population-based strategy; while not independently sufficient, the colon exhibit could complement other evidence-based approaches to CRC prevention and education. To date, however, most participants in inflatable-colon studies have been female (12–15). Additional research is needed to better understand the usefulness of this resource for CRC prevention and control among men. The objective of our study was to evaluate the influence of an inflatable colon as an educational tool to increase CRC knowledge and screening intent among men aged 18 to 75 years attending state fairs in 2 midwestern states.

## Methods

### Study participants

This observational study, which followed the Strengthening the Reporting of Observational Studies in Epidemiology (STROBE) reporting guidelines for cohort studies (www.strobe-statement.org), was conducted in summer 2023, during the final weekends of state fairs in 2 midwestern states. The Medical College of Wisconsin’s institutional review board approved all study procedures, marketing materials, and survey instruments before data collection (approval no. PRO47143). To encourage study participation, advertisements were posted at public community locations, on social media, via email, and on the study website, leading up to the events. To assist with recruitment and study implementation, research staff were recruited from community settings, including local universities, Craigslist, gastroenterology centers, the American Cancer Society, and social media platforms.

Individuals were eligible to participate if they self-identified as male, were aged 18 to 75 years, resided in state A (for site A) or state B (for site B), attended the state fair in their state of residence, and could read and speak English. Before participation, informed consent was obtained from all participants via an Apple iPad or cellphone by using the internet-based IRB-compliant PsychData survey system (Divergent Web Solutions, LLC). Participants could request access to preliminary study results and provide recommendations for future research and advocacy efforts via a community dialogue session held at a later time.

### Intervention

The Super Colon, an inflatable educational exhibit through which participants can walk, allows participants to closely observe models of normal and inflamed colon tissue, benign and malignant polyps, and invasive and metastatic CRC. Participants at each study site completed a presurvey, an unguided tour, and a postsurvey. After completing the postsurvey, participants were given a drawstring bag (with study logo on it and an ACS colorectal cancer brochure inside it) and an opportunity to enter a drawing for additional incentives such as gift cards, an iPad, or a television.

### Data collection

Data were collected through PsychData surveys completed on iPads or cellphones. We adapted our questions based on previously used survey items (14,17,19,20). The forced-choice surveys had 64 items (56 on the presurvey, 8 on the postsurvey). On average, study completion (presurvey, tour, and postsurvey) took 10 to 15 minutes. The surveys were administered in English. Staff were available to help read questionnaires to participants who needed assistance.

### Measures

The primary outcomes of interest were changes in CRC knowledge (actual and perceived) and behavioral intent to obtain CRC screening from baseline (presurvey) to intervention completion (postsurvey). Actual knowledge was defined as the comprehensive understanding and awareness of factual information, whereas self-perceived knowledge related to a participant’s own assessment of their understanding or familiarity with CRC. Actual knowledge was assessed by correct responses to 3 true-or-false statements in both surveys. The presurvey (but not the postsurvey) had this statement: “Men at average risk should have their first screening for colorectal cancer at age 35.” The correct answer is “false.” (The inflatable colon did not have information on age at first screening, and we observed that participants were wondering if they had missed the information and needed to walk through the inflatable colon again. Because of the confusion created by the item, we did not assess it at postsurvey.) The first statement assessed at both time points was, “If I have a family member with colorectal cancer, I am at a higher risk of having it too.” The correct answer is “true.” The second statement was, “Removing a polyp from my colon can prevent colorectal cancer.” The correct answer is “true.” The third question was, “Colorectal cancer always has symptoms that you can feel.” The correct answer is “false.”

Perceived knowledge was assessed with 3 items. One was the following true-or-false statement: “I know what a colon polyp is.” The second and third items were questions: 1) “How much do you feel you know about colorectal cancer now?” and 2) “How much do you feel you know about how colorectal cancer progresses now?” Response options were “a lot,” “some things,” and “nothing.”

Lastly, we assessed CRC screening intent with the question, “Do you plan to obtain colorectal cancer screening in the future?” Response options were 1) yes, in the next 6 months, 2) yes, in the next 7 months to 1 year, 3) yes, in 13 months to 2 years, 4) yes, sometime but not within 2 years, 5) no, but have considered getting screened, or 6) no, will not get screened. Participants were categorized as having screening intent if they chose options 1, 2, 3, or 4. 

We collected data on the following demographic characteristics in the presurvey: age, self-identified race and ethnicity, sexual orientation, relationship status, educational attainment, type of health insurance coverage, having a regular health care provider, personal and family history of CRC, and history of participation in CRC screening. The race and ethnicity variable was used to reflect membership in a societally imposed marginalized racial and ethnic group and as a proxy for systematic and structural racism. We combined responses for the 2 concepts of race and ethnicity, as recommended due to high nonresponse rates among Hispanic and Latino individuals when separate questions are used (21,22). Categories were combined for cell sizes of 10 or fewer participants.

### Statistical analysis

We used SAS version 9.4 (SAS Institute, Inc) to manage data and conduct our analysis in October 2023. We checked data through exploratory analysis statistics, including inspection for missing values and data-entry errors. Because less than 5% of participants were lost to follow-up (ie, did not complete the postsurvey), we excluded from analysis any participant with missing data for the outcomes of interest (ie, we used complete case analyses).

We generated descriptive statistics to examine the distribution of characteristics in the full study sample. We used US Census 2022 data (23) to compare the demographic characteristics of our study participants with the demographic characteristics of the population of men aged 18 to 75 years residing in the 2 states in which the state fairs were held (the population of interest). We used χ^2^ tests to examine differences in characteristics by study site and the association between selected demographics and behaviors (ie, knowledge and intentions) before entry into the Super Colon exhibit. We calculated the percentage of participants who responded correctly to the actual knowledge questions, the percentage of participants who indicated they knew what a colon polyp is, the percentage of participants who responded “a lot” or “some things” to the 2 items on perceived knowledge, and the percentage of participants who indicated they intended to be screened for CRC within the next 2 years in the presurvey and postsurvey and by study site. We used the McNemar test to examine differences in distributions from presurvey to postsurvey; a 2-sided *P* value <.05 was considered significant.

## Results

A total of 953 eligible participants completed the presurvey. The final sample comprised 940 men who finished both the presurvey and postsurvey (572 [60.8%] at site A; 368 [39.2%] at site B) (Figure). The largest proportion of participants self-reported their race and ethnicity as non-Hispanic White, sexual orientation as heterosexual, and relationship status as either married (site A) or never married (site B) (Table 1). Many participants had completed at least some college, had private health insurance, and reported having a health care provider whom they saw regularly. Slightly more than half of the participants reported having completed a stool-based test or an examination-based test. Most men aged 45 or older had been previously screened for CRC with either a stool-based test or an examination-based test. Approximately 1 in 8 participants had walked through an inflatable colon previously.

**Figure:**
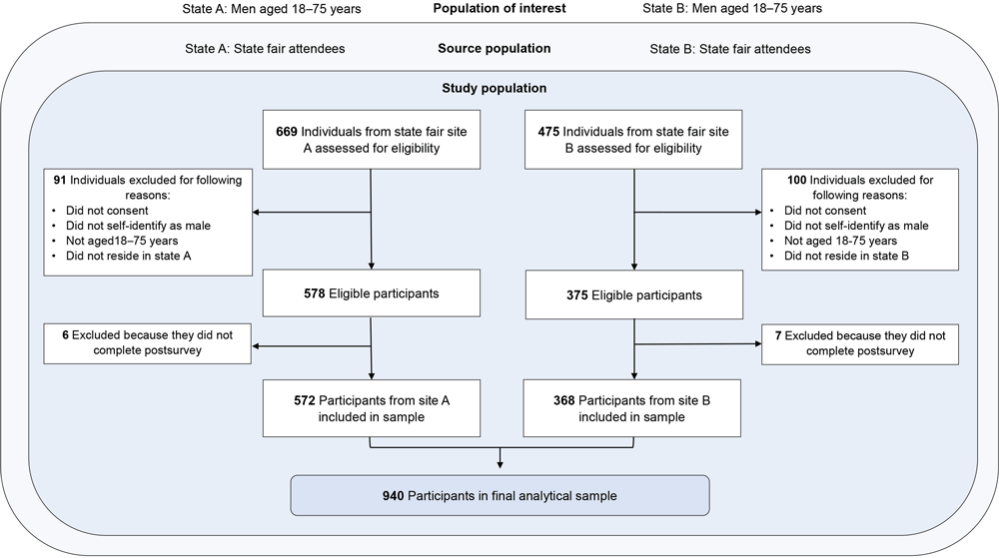
Flowchart showing how men aged 18 to 75 years were selected to participate in a study of colorectal screening knowledge and intent at state fairs in 2 midwestern states, 2023.

**Table 1:** Characteristics of Study Sample, Men Aged 18–75 Years at 2 State Fairs in Midwestern States A and B (N = 940), by Site, 2023^a^

Characteristics	Total	Study site A	Study site B	*P* value^b^
**No. (%)**	940 (100.0)	572 (60.8)	368 (39.2)	—
**Demographic characteristics**				
**Age, mean (SD), y**	40.9 (15.1)	41.5 (15.6)	39.9 (14.4)	.10
**Age group, y**				
18–30	312 (33.2)	192 (33.6)	120 (32.6)	.009
31–45	269 (28.6)	149 (26.0)	120 (32.6)	
46–59	218 (23.2)	129 (22.6)	89 (24.2)	
60–75	141 (15.0)	102 (17.8)	39 (10.6)	
**Self-identified race and ethnicity**				
Hispanic or Latino	72 (7.7)	28 (4.9)	44 (12.0)	<.001
Non-Hispanic Asian	50 (5.2)	39 (6.8)	11 (3.0)	
Non-Hispanic Black	81 (8.6)	37 (6.5)	44 (12.0)	
Non-Hispanic White	676 (71.9)	431 (75.4)	245 (66.6)	
Non-Hispanic multiracial	25 (2.6)	14 (2.4)	11 (3.0)	
Non-Hispanic Other^c^	36 (3.8)	23 (4.0)	13 (3.5)	
**Sexual orientation**				
Straight or heterosexual	853 (90.7)	513 (89.7)	340 (92.4)	.16
Nonheterosexual	87 (9.3)	59 (10.3)	28 (7.6)	
**Relationship status**				
Married	469 (49.9)	302 (52.8)	167 (45.4)	.03
Divorced, widowed, or separated	46 (4.9)	22 (3.8)	24 (6.5)	
Never married	425 (45.2)	248 (43.4)	177 (48.1)	
**Educational attainment**				
High school or less	153 (16.3)	64 (11.2)	89 (24.2)	<.001
Some college	244 (26.2)	122 (21.4)	122 (33.8)	
Bachelor’s degree	324 (34.8)	229 (40.1)	95 (26.3)	
Master’s degree or more	219 (23.5)	157 (27.5)	62 (17.2)	
**Health insurance coverage**				
Private	717 (76.3)	452 (79.0)	265 (72.0)	.01
Public (Medicare, Medicaid, Tricare)	141 (15.0)	83 (14.5)	58 (15.8)	
Uninsured	82 (8.7)	37 (6.5)	45 (12.2)	
**Has a regular health care provider**				
Yes	652 (69.4)	380 (66.4)	272 (73.9)	.02
No	288 (30.6)	192 (33.6)	96 (26.1)	
**Personal and family history of cancer**				
**Family history of cancer**				
Yes	424 (45.1)	273 (47.7)	151 (41.0)	.12
No	423 (45.0)	247 (43.2)	176 (47.8)	
Not sure	93 (9.9)	52 (9.1)	41 (11.1)	
**Family history of colorectal cancer**				
Yes	128 (13.6)	86 (15.0)	42 (11.4)	.09
No	691 (73.5)	421 (73.6)	270 (73.4)	
Not sure	121 (12.9)	65 (11.4)	56 (15.2)	
**Diagnosed colorectal cancer**				
Yes	13 (1.4)	7 (1.2)	6 (1.6)	.60
No	927 (98.6)	565 (98.8)	362 (98.4)	
**History of participation in CRC screening**				
**Completed stool-based test**				
Yes	255 (27.1)	123 (21.5)	132 (35.9)	<.001
No	685 (72.9)	449 (78.5)	236 (64.1)	
**Completed examination-based test**				
Yes	394 (41.9)	249 (43.5)	145 (39.4)	.21
No	546 (58.1)	323 (56.5)	223 (60.6)	
**Completed stool-based test or examination-based test among those aged 45–75 years**				
Either test	333 (86.7)	215 (88.8)	118 (83.1)	.11
Neither test	51 (13.3)	27 (11.2)	24 (16.9)	
**Have you walked through an inflatable-colon exhibit before today?**				
Yes	120 (12.8)	70 (12.2)	50 (13.6)	.55
No	820 (87.2)	502 (87.8)	318 (86.4)	

Abbreviation: CRC, colorectal cancer.

a All values are number (percentage) unless otherwise indicated. Data were collected at baseline (before entry into the Super Colon exhibit) only.

b Determined by χ^2^ test; *P* < .05 considered significant.

c Includes Native Hawaiian or Pacific Islander, American Indian or Alaska Native, and “Other race.”

A comparison of demographic characteristics at the 2 study sites showed significant differences by age group, race and ethnicity, relationship status, educational attainment, type of health insurance coverage, having a regular health care provider, and completion of a stool-based test (Table 1). Compared with participants at site A, participants at site B were less likely to be non-Hispanic White (75.4% vs 66.6%), married (52.8% vs 45.4%), to have completed college (bachelor’s degree, 40.1% vs 26.3%; master’s degree, 27.5% vs 17.2%), to have private health insurance (79.0% vs 72.0%), and not to have a regular health care provider (33.6% vs 26.1%). Participants at site B were more likely than participants at site A to report ever completing a stool-based test (35.9% vs 21.5%).

In a comparison of the demographic characteristics of our study sample with 2022 US Census data for men aged 18 to 75 years residing in the 2 midwestern states, we found that at both study sites, participants aged 60 to 75 years (site A: 17.8% vs 24.5%; site B: 10.6% vs 25.7%) and participants with high school or less were less frequent in our study samples than in the US Census populations (site A: 11.2% vs 31.3%; site B: 24.2% vs 38.8%). Similarly, participants aged 18 to 30 years (site A: 33.6% vs 24.0%; site B: 32.6% vs 24.0%), participants who never married (site A: 43.4% vs 34.7%; site B: 48.1% vs 35.4%), and participants with a bachelor’s degree or more (site A: 67.6% vs 34.8%; site B: 43.5% vs 28.4%) were more frequent in our study samples than in the US Census populations (Table 2). In addition, at study site B, participants who self-identified as non-Hispanic Black (12.0% vs 5.4%) or Hispanic/Latino (12.0% vs 6.8%) and participants who had no health insurance (12.2% vs 7.6%) were more frequent in our study sample than in the US Census populations.

**Table 2:** Demographic Characteristics of Study Sample, Men Aged 18–75 Years at 2 State Fairs in Midwestern States A and B (N = 940), by Site, Compared With Population of Interest, 2023

Characteristic	Site A		Site B	
	Study sample	State A^a^	Study sample	State B^a^
**Total**	572	2,063,254	368	2,155,860
**Age group, y**				
18–30	192 (33.6)	495,687 (24.0)	120 (32.6)	516,560 (24.0)
31–45	149 (26.0)	590,521 (28.6)	120 (32.6)	565,491 (26.2)
46–59	129 (22.6)	472,077 (22.9)	89 (24.2)	519,778 (24.1)
60–75	102 (17.8)	504,969 (24.5)	39 (10.6)	554,031 (25.7)
**Self-identified race and ethnicity**				
Hispanic/Latino/Spanish^b^	28 (4.9)	111,640 (5.4)	44 (12.0)	145,567 (6.8)
Non-Hispanic Black	37 (6.5)	136,322 (6.6)	44 (12.0)	115,712 (5.4)
Non-Hispanic White	431 (75.4)	1,610,606 (78.1)	245 (66.6)	1,745,683 (81.0)
Non-Hispanic Other	76 (13.3)	204,686 (9.9)	35 (9.5)	148,898 (6.9)
**Relationship status**				
Married	302 (52.8)	1,115,152 (54.0)	167 (45.4)	1,121,536 (52.0)
Divorced, widowed or separated	22 (3.8)	232,210 (11.2)	24 (6.5)	270,048 (12.5)
Never married	248 (43.4)	715,892 (34.7)	177 (48.1)	764,276 (35.4)
**Educational attainment**				
High school or less	64 (11.2)	646,286 (31.3)	89 (24.2)	837,625 (38.8)
Some college	122 (21.4)	664,737 (32.2)	122 (33.8)	681,997 (31.6)
Bachelor’s degree	229 (40.1)	490,685 (23.8)	95 (26.3)	415,900 (19.3)
Master’s degree or more	157 (27.5)	227,583 (11.0)	62 (17.2)	196,973 (9.1)
**Health insurance coverage**				
Insured	535 (93.5)	1,928,916 (93.5)	323 (87.8)	1,991,826 (92.4)
Uninsured	37 (6.5)	134,338 (6.5)	45 (12.2)	164,034 (7.6)

a Data source: US Census Bureau (23). All values are number (percentage) unless otherwise indicated. Data for study participants were collected at baseline (before entry into the Super Colon exhibit) only.

b US Census Bureau data included the term “Spanish.”

### Knowledge and intentions

Before entering the Super Colon, approximately one-third of participants correctly answered the question about when men at average risk should initiate CRC screening (Table 3). However, most (90.1%) knew that a family history of CRC increases their own CRC risk. Participants of screening age (ie, aged 45–75 y), compared with participants aged 45 years or younger, had significantly greater actual CRC knowledge but less self-perceived knowledge and were more likely to intend to be screened within 2 years.

**Table 3:** Actual Knowledge and Self-Perceived Knowledge About CRC and Screening Intention for CRC Before Viewing an Inflatable Colon, Men Aged 18–75 Years at 2 State Fairs in the Midwest (N = 940), 2023^a^

Characteristic	Actual knowledge (answered correctly)				Self-perceived knowledge			Intend to be screened^i^
	Item 1 (age at first screen)^b^	Item 2 (family risk)^c^	Item 3 (polyp removal)^d^	Item 4 (feeling symptoms)^e^	Item 1 (know what a polyp is)^f^	Item 2 (know about CRC)^g^	Item 3 (know about CRC progression)^h^	
**No. (%) of participants**	335 (35.6)	847 (90.1)	769 (81.8)	827 (88.0)	705 (75.0)	707 (75.2)	609 (64.8)	740 (78.7)
**Age group, y**								
≤45	160 (27.5)	527 (90.7)	452 (77.8)	495 (85.2)	376 (64.7)	519 (89.3)	535 (92.1)	409 (70.4)
>45	175 (48.8)	320 (89.1)	317 (88.3)	332 (92.5)	329 (91.6)	284 (79.1)	301 (83.8)	331 (92.2)
*P* value^j^	<.001	.44	<.001	<.001	<.001	<.001	<.001	<.001
**Educational attainment**								
Some college or less	105 (26.4)	342 (86.2)	302 (76.1)	335 (84.4)	263 (66.2)	354 (89.2)	361 (90.9)	292 (73.6)
Bachelor’s degree or more	230 (42.4)	505 (93.0)	467 (86.0)	492 (90.6)	442 (81.4)	449 (82.7)	475 (87.5)	448 (82.5)
*P* value^j^	<.001	<.001	<.001	.004	<.001	.005	.10	<.001
**Has a regular health care provider**								
Yes	247 (37.9)	583 (89.4)	547 (83.9)	582 (89.3)	511 (78.4)	534 (81.9)	561 (86.0)	534 (81.9)
No	88 (30.6)	264 (91.7)	222 (77.1)	245 (85.1)	194 (67.4)	269 (93.4)	275 (95.5)	206 (71.5)
*P* value^j^	.03	.29	.01	.07	<.001	<.001	<.001	<.001
**Self-identified race and ethnicity**								
Non-Hispanic White	268 (39.6)	622 (92.0)	572 (84.6)	621 (91.9)	534 (79.0)	582 (86.1)	607 (89.8)	556 (82.2)
All other races	67 (25.4)	225 (85.2)	197 (74.6)	206 (78.0)	171 (64.8)	221 (83.7)	229 (86.7)	184 (69.7)
*P* value^j^	<.001	.002	<.001	<.001	<.001	.35	.18	<.001
**Study site**								
A	235 (41.1)	526 (92.0)	472 (82.5)	515 (90.0)	437 (76.4)	484 (84.6)	510 (89.2)	469 (82.0)
B	100 (27.2)	321 (87.2)	297 (80.7)	312 (84.8)	268 (72.8)	319 (86.7)	326 (88.6)	271 (73.6)
*P* value^j^	<.001	.02	.48	.02	.22	.38	.78	.002
**Ever completed a stool-based test or an examination-based test or both**								
Yes	189 (36.9)	454 (88. 7)	440 (85.9)	445 (86.9)	441 (86.1)	416 (81.2)	436 (85.2)	435 (85.0)
No	146 (34.1)	393 (91.8)	329 (76.9)	382 (89.2)	264 (61.7)	387 (90.4)	400 (93.5)	305 (71.3)
*P* value^j^	.37	.11	<.001	.27	<.001	<.001	<.001	<.001
**Relationship status**								
Married	196 (41.8)	430 (91.7)	409 (87.2)	432 (92.1)	396 (84.4)	387 (82.5)	402 (85.7)	400 (85.3)
Not married	139 (29.5)	417 (88.5)	360 (76.4)	395 (83.9)	309 (65.6)	416 (88.3)	434 (92.1)	340 (72.2)
*P* value^j^	<.001	.11	<.001	<.001	<.001	.01	.002	<.001

Abbreviation: CRC, colorectal cancer.

a All values are number (percentage) of participants who answered correctly to items on actual knowledge or who answered as indicated to items on self-perceived knowledge or intent to be screened.

b The true–false item was “Men at average risk should have their first screening for CRC at age 35?” The correct answer is “false.”

c The true–false item was “If I have a family member with CRC, I am at a higher risk of having it too.” The correct answer is “true.”

d The true–false item was “Removing a polyp from my colon can prevent CRC.” The correct answer is “true.”

e The true–false item was “CRC always has symptoms that you can feel.” The correct answer is “false.”

f Response of “true” to the true–false item, “I know what a colon polyp is.”

g Response of “a lot” or “some things” to question, “How much do you feel you know about CRC now?” Response options were “a lot,” “some things,” or “nothing.”

h Response of “a lot” or “some things” to question, “How much do you feel you know about how CRC progresses now?” Response options were “a lot,” “some things,” or “nothing.”

i Response of yes, regardless of time, to question, “Do you plan to obtain colorectal cancer screening in the future?”. Response options were yes, in the next 1) 6 months, 2) 7 months to 1 year, 3) 13 months to 2 years, 4) sometime but not within 2 years; or no, 5) but have considered getting screened, or 6) will not get screened.

j Determined by χ^2^ test.

We observed significant differences in responses to the knowledge and intent items by educational attainment on the presurvey. Participants with some college or less, compared with participants with a bachelor’s degree or more, had greater self-perceived knowledge of CRC on the presurvey (for 2 of the 3 items) but were less likely to answer the knowledge items correctly. Participants with a regular health care provider were more likely than participants without one to know the recommended age to start CRC screening, that removing polyps can prevent CRC, to have lower self-perceived CRC knowledge (for 2 of the 3 items), and to intend to be screened within the next 2 years. Participants who had never completed a blood-based test or an examination-based test were more likely than those who had completed one to have greater self-perceived knowledge (for 2 of the 3 items). 

We found significant improvements at both sites from presurvey to postsurvey in knowing that removing a polyp can prevent CRC; in self-perceived knowledge about what a colon polyp is, what CRC is, and how CRC progresses; and in intention to be screened within next 2 years (Table 4). At Site B, from presurvey to postsurvey, participants significantly decreased in knowledge that CRC does not always have symptoms that can be felt (from 84.8% to 79.9%). In the postsurvey, 94.5% of participants agreed that an inflatable colon is an effective tool for teaching people about CRC.

**Table 4:** Actual Knowledge and Self-Perceived Knowledge About CRC and Screening Intention for CRC Screening Before and After Viewing an Inflatable Colon, Men Aged 18–75 Years at 2 State Fairs in Midwestern States A and B (N = 940), 2023^a^

Item	Total sample			Site A			Site B		
	Pre	Post	*P* value^b^	Pre	Post	*P* value^b^	Pre	Post	*P* value^b^
**Actual knowledge**									
Item 1 (age at first screening)^d^	35.6	—c	—	41.0	—c	—	27.2	—c	—
Item 2 (family risk)^e^	90.1	89.9	.85	92.0	90.6	.28	87.2	88.9	.43
Item 3 (polyp removal)^f^	81.8	91.3	<.001	82.5	92.7	<.001	80.7	89.1	<.001
Item 4 (feeling symptoms)^g^	88.0	85.6	.05	90.0	89.3	.62	84.8	79.9	.02
**Self-perceived knowledge**									
Answered “true” to “I know what a colon polyp is.”	75.0	96.2	<.001	76.4	96.0	<.001	72.8	96.5	<.001
“How much do you feel you know about CRC now?”^h^									
A lot	14.6	33.6	<.001	15.4	33.2	<.001	13.3	34.2	<.001
Some things	60.6	62.8		60.0	64.2		61.7	60.6	
“How much do you feel you know about how CRC progresses now?”^h^									
A lot	11.1	36.7	<.001	10.8	37.2	<.001	11.4	35.9	<.001
Some things	53.7	60.2		53.7	60.0		53.8	60.6	
**Intend to be screened, %^i^**	78.7	86.1	<.001	82.0	87.4	<.001	73.6	84.0	<.001

Abbreviation: —, does not apply; CRC, colorectal cancer.

a All values are number (percentage) of participants who answered correctly to items on actual knowledge or who answered as indicated to items on self-perceived knowledge or intent to be screened.

b Determined by McNemar test.

c Not assessed at postsurvey because the inflatable colon did not have information on age at first screening, and we observed that participants were wondering if they had missed the information and needed to walk through the inflatable colon again.

d The true–false item was “Men at average risk should have their first screening for CRC at age 35?” The correct answer is “false.” The item was not included on the postsurvey.

e The true–false item was “If I have a family member with CRC, I am at a higher risk of having it too.” The correct answer is “true.”

f The true–false item was “Removing a polyp from my colon can prevent CRC.” The correct answer is “true.”

g The true–false item was “CRC always has symptoms that you can feel.” The correct answer is “false.”

h Response options were “a lot,” “some things,” or “nothing.”

i Percentage of participants who answered yes, regardless of time, to question, “Do you plan to obtain colorectal cancer screening in the future?” Response options were yes, in the next 1) 6 months, 2) 7 months to 1 year, 3) 13 months to 2 years, 4) sometime but not within 2 years; or no, 5) but have considered getting screened, or 6) will not get screened.

## Discussion

To our knowledge, this study is the first to evaluate the effectiveness of an inflatable colon as an educational tool to increase CRC knowledge and screening intent among men in a state fair setting. In our sample of 940 men aged 18 to 75 years, touring the inflatable colon led to significant improvements in knowledge and screening intent. Apart from the question on when men should have their first CRC screening, our study sample at baseline demonstrated relatively high actual CRC knowledge. Compared with a similar study conducted in Alaska and Canada that used similar knowledge questions, our study demonstrated less knowledge among participants about the appropriate age to start CRC screening (35.6% vs 65.0%) and that CRC does not always have symptoms that can be felt (88.0% vs 92.0%) (14). Conversely, participants in our study demonstrated more knowledge about family CRC risk (90.1% vs 88.0%), with no meaningful difference in understanding the role of polyp removal in preventing CRC (81.8% vs 81.0%) (14).

Among participants with educational attainment of some college or less, 90.9% felt they knew “some things” or “a lot” about how CRC progresses, compared with 87.5% of those with higher educational attainment. However, participants with some college or less had a lower prevalence of correct answers on actual knowledge statements compared with those with higher educational attainment. We observed similar patterns among other subgroups (ie, an inverse relationship between perceived knowledge and actual knowledge) including among participants of screening-eligible age, CRC screening-participation history, and marital status. Subgroups with higher CRC knowledge also had a higher prevalence of screening intent, supporting previous findings that associated higher education levels with higher CRC screening participation (24–26). Participants aged 45 years or younger and racial and ethnic minority men had less knowledge and screening intent than their older and non-Hispanic White counterparts. Given the prominent health disparities affecting racial and ethnic minority populations and the projected increase in CRC-related deaths among adults aged less than 50 years by 2030, heightened research efforts and national funding directed to improving CRC knowledge and screening intent in both the under-45 and racial and ethnic minority populations are imperative (11).

Knowledge and beliefs are important factors that enable health behaviors such as participation in early detection screening. In line with other literature (12–15,17,18,27,28), we observed significant improvements in participants’ actual and self-perceived knowledge about colon polyps and screening intent after they completed the inflatable-colon tour. Our findings support the effectiveness of community education and outreach events in promoting CRC knowledge and awareness. Specifically, our study highlights the value of self-guided tours of an inflatable colon as a low-resource–intensive intervention at such events.

Large recreational gatherings such as state fairs can attract populations that might not otherwise have ready access to or familiarity with cancer prevention and control services (11). These events may also reach groups that differ according to demographic characteristics (eg, education). These differences may be related to the higher rate of screening participation in our sample compared with that observed elsewhere (29–32). Men aged 60 to 75 years and men with a high school education or less were less frequently represented at the 2 study sites than they were in our population of interest. In contrast, men aged 18 to 30 years, never-married men, and men with higher educational attainment were more frequently represented in our study samples than in the population of interest. Specifically, at site B, participants who self-identified as non-Hispanic Black and Hispanic/Latino and participants who were uninsured were more frequently represented in our study sample than in the population of interest. Because the current body of evidence is inconclusive about whether special events effectively enhance CRC screening rates among men, future research is warranted.

### Limitations

Although this study contributes to the literature on the effectiveness of using an inflatable colon to improve CRC knowledge, the use of a descriptive epidemiologic approach has limitations related to measurement accuracy, potential selection bias, and the lack of a control group (33,34). Our use of self‐reported data may have increased susceptibility to misclassification (ie, information bias). Although we believe our use of self-reported data did not significantly affect the collection of demographic data, because the use of self-reported race and ethnicity is currently considered the gold standard and less likely to result in misclassification (35), social desirability bias may have influenced our outcomes of interest (knowledge and intent). To address this concern, we incorporated proactive measures into the study design, including collecting no personal identifying information and having at least 1 research staff member nearby while participants completed the presurveys and postsurveys. Although the reliability and validity for actual knowledge scales and CRC screening intent scales have been reported elsewhere (16,36), further assessment of the psychometric properties of our questions that assessed perceived CRC knowledge postintervention is necessary (17). Of note, social desirability bias would likely have affected responses to both surveys, ensuring consistency in our conclusions. However, the alignment of our findings with existing literature reinforces our confidence in them.

Self-selection can bias descriptive studies when study participation is associated with the outcome. Using convenience samples, especially when participation involves opting in, often leads to study samples that differ from the population of interest in terms of sociodemographic factors and health behaviors. While our study sample differed slightly from the population of interest, it may have been more inclined to make behavioral changes; for example, more willing to participate in CRC screening because of high educational attainment. Additionally, our approach of mandating responses to all questions, while eliminating the problem of missing data, may have had the unintended consequence of causing individuals to exit the survey when they were unable to skip questions they preferred not to answer (ie, none of their data were saved).

### Strengths

Despite these limitations, our study demonstrates several strengths. Descriptive studies that precisely estimate a parameter of interest and are easily interpretable to clinicians and policymakers contribute substantially to the advancement of public health. Our study adds to the literature on inflatable colons as a CRC education tool (14,15). Whereas previous studies relied on data from 1 geographical region, ours used data from participants with diverse sociodemographic backgrounds in 2 midwestern states. Our study’s inability to determine whether reported CRC screening intent translated into screening completion presents an avenue for exploration in future research. Community engagement, partnerships, and relationship building were additional anecdotal study benefits.

### Conclusions

Our research highlights the importance of community-based educational programs in promoting CRC screening and increasing men’s participation in research. It confirms that the inflatable colon serves as an effective educational tool for raising CRC knowledge and encouraging men to undergo early-detection screening. These findings can inform the development of future health promotion initiatives tailored to men and contribute to our understanding of the effect of community education and outreach events focusing on men.
